# Supplementary material for: Approaches for Memristive Structures Using Scratching Probe Nanolithography: Towards Neuromorphic Applications
Source: Nanomaterials (Basel). 2023 May 9;13(10):1583. doi: 10.3390/nano13101583 (PMC10223422; doi:10.3390/nano13101583)
Supplement: Supplementary file 1 [file nanomaterials-13-01583-s001.zip › nanomaterials-2348941-supplementary.pdf]

nanomaterials

Article

Approaches for memristive structures using scratching probe nanolithography: towards neuromorphic applications

Roman V. Tominov[1,2], Zakhar E. Vakulov[1], Vadim I. Avilov[1], Ivan A. Shikhovtsov[1], Vadim I. Varganov[1], Victor B. Kazantsev[1,3] *, Lovi Raj Gupta[1,4], Chander Prakash[1,5], Vladimir A. Smirnov[1,2]

Research Laboratory Neuroelectronics and Memristive Nanomaterials (NEUROMENA Lab), Institute of Nanotechnologies, Electronics and Electronic Equipment Engineering, Southern Federal University, Taganrog 347922, Russia; tominov@sfedu.ru (R.V.T.); zvakulov@sfedu.ru (Z.E.V.); avilovvi@sfedu.ru (V.I.A.); shihovcov@sfedu.ru (I.A.S.); varganov@sfedu.ru (V.I.V.); kazantsev@neuro.nnov.ru (V.B.K.); lovi.raj@lpu.co.in (L.R.G.); chander.mechengg@gmail.com (C.P.)

2 Department of Radioelectronics and Nanoelectronics, Institute of Nanotechnologies, Electronics and Electronic Equipment Engineering, Southern Federal University, Taganrog 347922, Russia

3 Institute of Biology and Biomedicine, National Research Lobachevsky State University of Nizhny Novgorod, Nizhny Novgorod 603950, Russia

4 Division of Research and Development, Lovely Professional University, Phagwara 144411, Panjab, India

5 School of Mechanical Engineering, Lovely Professional University, Phagwara 144411, Panjab, India

* Correspondence: vasmirnov@sfedu.ru

Received: date; Accepted: date; Published: date

The analysis of statistical results obtained for the depth (Table S1) shows that for the normal load, the difference between the means is statistically significant (at the 95% level), and the contribution to the total sum of squares is 79.584%; for the scratch distance, the difference between the means is statistically significant, and the contribution to the total sum of squares is 4.560%; for the probe speed, the difference between the means is statistically insignificant; for the probe direction, the difference between the mean is insignificant. Thus, the greatest influence on the depth is, as would be expected, the normal load. The scratch distance and probe direction have a smaller influence compared to the normal load, and the probe speed within this model has almost no effect on the depth.

Table S1. ANOVA for the scratch depth results.

	Degrees of Freedom	Sum of Squares	Mean Square	F-ratio	P-value	Share of contribution to the total sum of squares (%)
A (Normal load)	2	905.540	452.770	401.867	0.035251	79.584
B (Scratch distance)	2	51.887	25.943	23.027	0.145782	4.560
C (Probe speed)	2	10.007	5.003	4.441	0.318116	0.879
D (Probe direction)	1	169.280	169.280	150.249	0.051822	14.877
Error	1	1.127	1.127			0.100
Total	8	1137.840				100

The analysis of statistical results obtained for the surface roughness (Table S2) shows that for the normal load, the difference between the means is statistically significant (at the 95% level), and the contribution to the total sum of squares is 52.050%; for the scratch distance, the difference between the means is statistically significant, and the contribution to the total sum of squares is 23.682%; for the probe speed, the difference between the means is statistically insignificant; for the probe direction, the difference between the means is statistically significant, and the contribution to the total sum of squares is 9.005%. Thus, the normal load has the greatest influence on the surface roughness. The scratch distance and the probe direction have less influence compared to the normal load, and the probe speed has a negligible influence.

Table S2. ANOVA for the surface roughness results.

	Degrees of Freedom	Sum of Squares	Mean Square	F-ratio	P-value	Share of contribution to the total sum of squares (%)
**A (Normal load)**	2	70.3356	35.1678	1.87795	0.458547	52.050
**B (Scratch distance)**	2	32.0022	16.0011	0.85446	0.607579	23.682
**C (Probe speed)**	2	1.8956	0.9478	0.05061	0.952933	1.402
**D (Probe direction)**	1	12.1689	12.1689	0.64982	0.568081	9.005
**Error**	1	18.7267	18.7267			13.861
**Total**	8	135.1289				100
